# Non-prescription cold and flu medication-induced transient myopia with uveal effusion: case report

**DOI:** 10.1186/s12886-019-1137-7

**Published:** 2019-06-26

**Authors:** Rui Zeng, Yun-peng Li, Chun-li Chen, Ya-qian Huang, Hao Lian, Yu-zhang Hu, Jia-song Yang

**Affiliations:** 1 Department of Ophthalmology, Yancheng Aier Eye Hospital, Yancheng, 224000 China; 2 Aier School of Ophthalmology, Central South University, Changsha, 410000 China; 3 Department of Ophthalmology, Apex Eye Hospital, Zhumadian, 463000 Henan Province China; 4 Department of Ophthalmology, Tianjin Medical University Eye Hospital, Tianjin, 30000 China; 5 Vitreous and Retinal Department, Chengdu Aidi Eye Hospital, Chengdu, 610000 Sichuan Province China; 6 Department of Pediatric Ophthalmology, Weiernuo Pediatric Clinic, Shanghai, 200050 China

**Keywords:** Uveal effusion, Acute angle-closure glaucoma, Non-prescription cold and flu medication

## Abstract

**Background:**

To report a case of non-prescription cold and flu medication-induced transient myopia with uveal effusion.

**Case presentation:**

Bilateral high intraocular pressure, shallow anterior chambers, uveal effusion, and a myopic shift were encountered in a 39-year-old Chinese male 1 night after taking a non-prescription flu medicine three times than the recommended dose. Ultrasound biomicroscopy (UBM) showed bilateral ciliochoroidal effusions, disappearance of the ciliary sulcus, closure of the angle of the anterior chamber, and anterior displacement of the lens-iris diaphragm. Treatment with aqueous suppressants was given. Within a week, the uncorrected vision restored, and the myopia had disappeared. UBM revealed major resolution of the ciliochoroidal effusions in both eyes, deepening of the anterior chamber, return of the lens-iris diaphragm to a more posterior position.

**Conclusions:**

Overdose of non-prescription cold and flu medication may cause bilateral uveal effusions inducing acute angle-closure glaucoma and acute myopia.

## Background

Drug-induced acute angle-closure glaucoma and uveal effusion have been occasionally reported [1]. Most of the drugs causing this condition are concentrated in antihypertensive drugs and antiepileptic drugs [2–4]. The use of over-the-counter (OTC) cold medicines is huge worldwide, and we present a case of transient bilateral myopic shift secondary to uveal effusion that occurred after taking OTC cold medicine.

## Case presentation

A 39-year old Chinese male patient transferred to our hospital for sudden onset of blurred vision in both eyes for 1 day. The patient went to sleep after taking the flu medicine (combination of paracetamol 250 mg, caffein 15 mg, atificial cow-bezoar 10 mg and chlorphenamine 1 mg) three times the recommended dose on the previous night. On the next morning, the vision of both eyes decreased significantly. There was no prior history of ocular disease or myopia. Other medical history was insignificant rather than hypertension was diagnosed more than 1 year ago. However, the patient denied taking antihypertensive drugs and any other drugs.

**Fig. 1:**
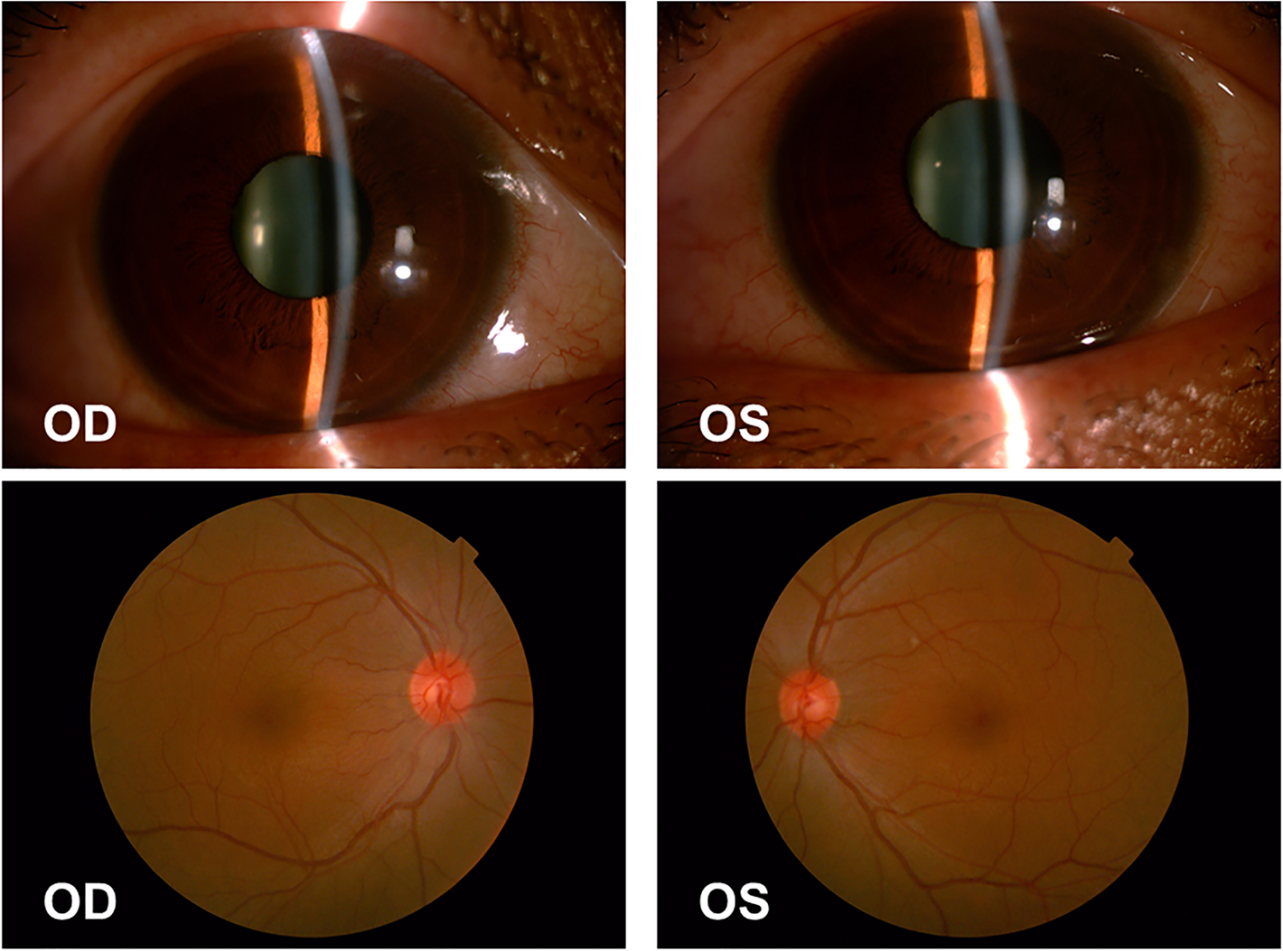
The anterior segment and fundus photograph showed the transparent corneas, shallow anterior chamber, round pupils and relatively normal fundus

**Fig. 2:**
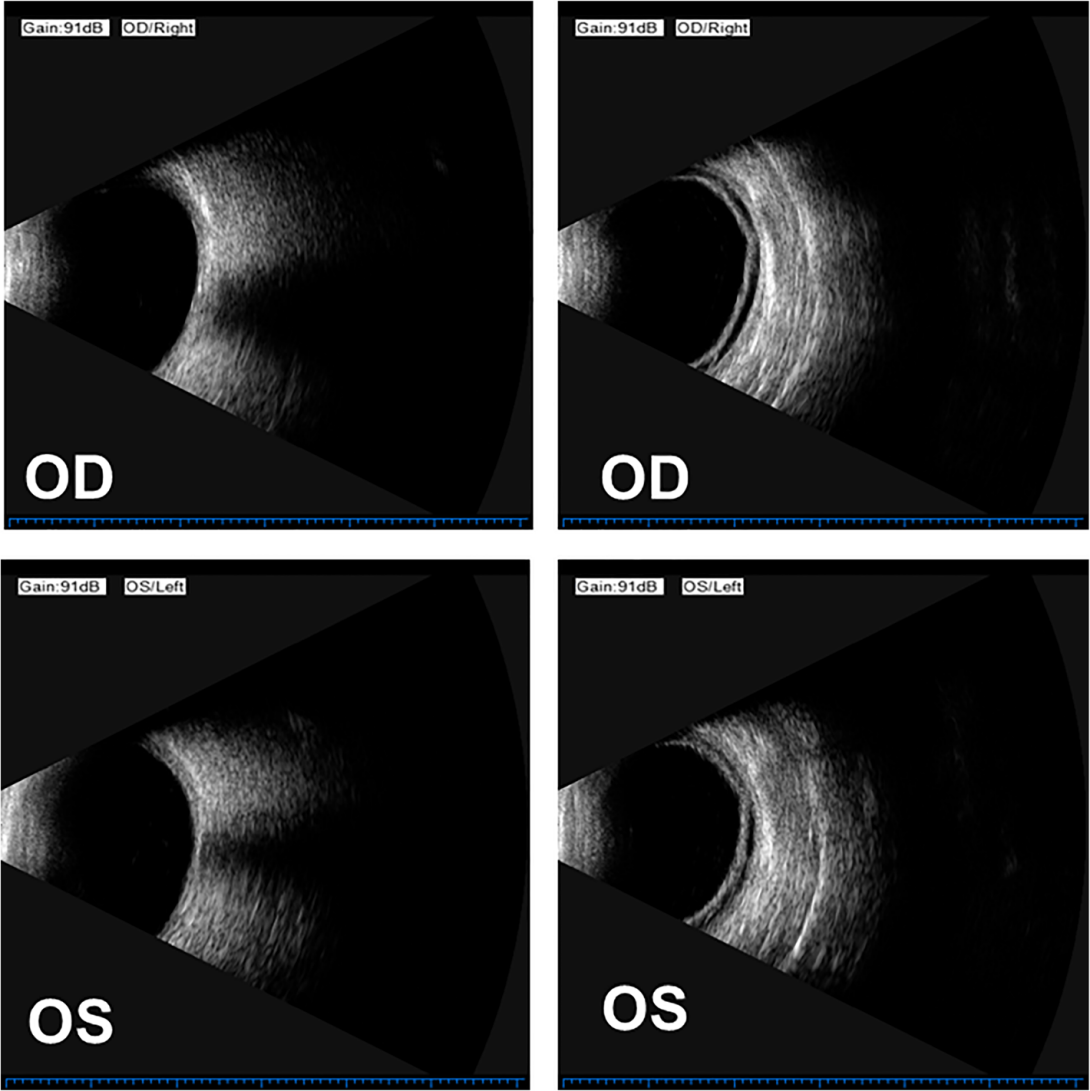
Ultrasonography showed that there were no special findings in vitreous cavity and posterior wall of the ball in both eyes, but the highly reflective band was separated from the wall in the periphery

**Fig. 3:**
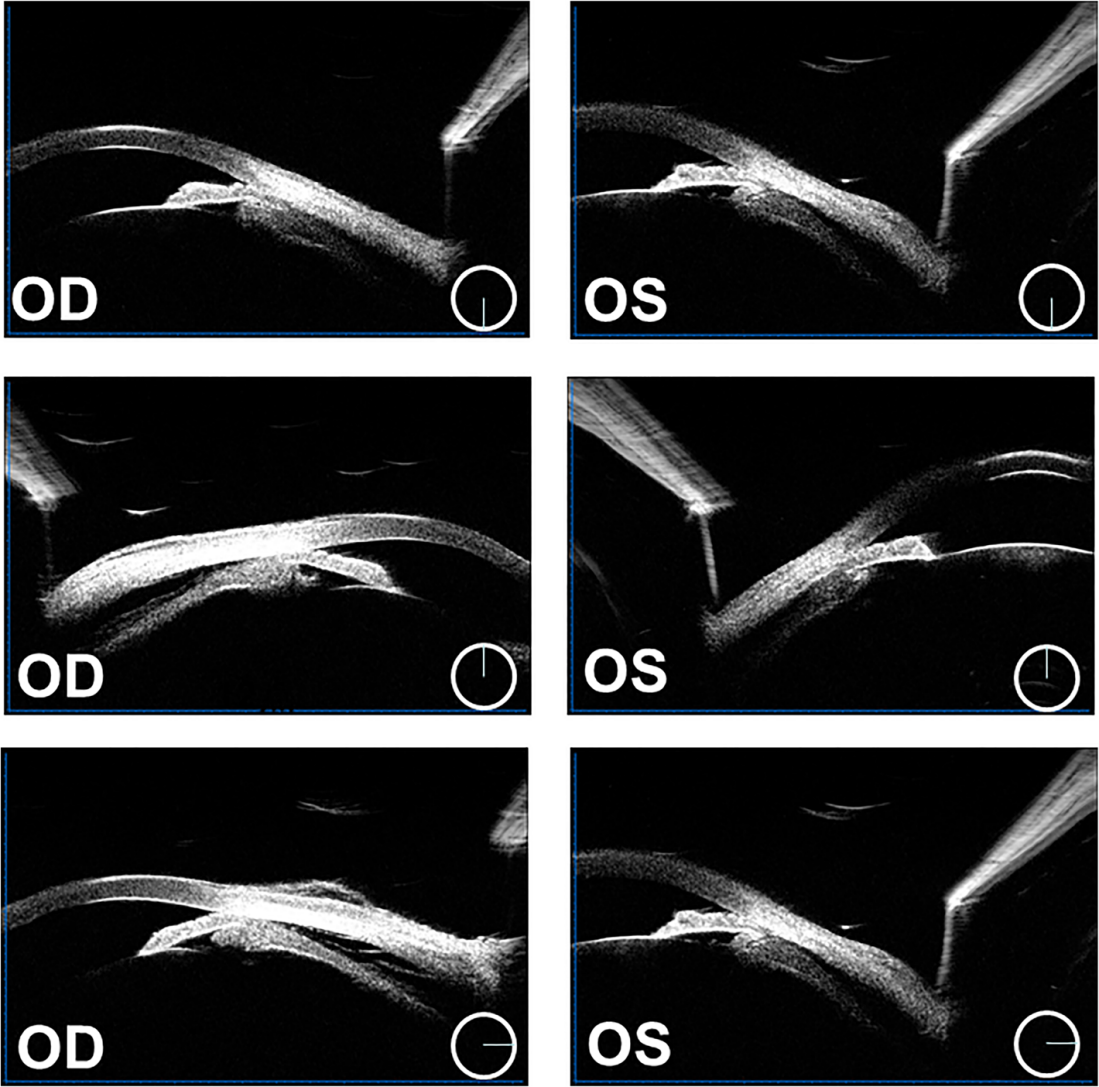
UBM showed bilateral ciliochoroidal effusions, disappearance of the ciliary sulcus, closure of the angle of the anterior chamber, and anterior displacement of the lens-iris diaphragm

Ocular examination revealed uncorrected vision of 20/400 in both eyes. Vision was corrected to 20/100 OD and 20/60 OS with − 4.50 OD and − 7.00 OS. Intraocular pressure (IOP) was 54 mmHg OD and 55 mmHg OS. Pupillary reactions were sluggish but present. Slit lamp examination revealed mild hyperemia of the bulbar conjunctivas, the cornea of both eyes was still transparent, shallow anterior chambers, the pupils were round, about 3 mm in diameter, and no other obvious abnormalities were found under the small pupil (Fig. 1). The central anterior chamber depth measured by Lenstar (Haag-Streit AG) was 1.97 mm in the right eye and 2.05 mm in the left eye. Lens thickness was 4.43 mm OD and 4.40 mm OS. OCT (Optovue, Inc., Freemont, CA) showed no obvious abnormality in macular region. Parapapillary OCT showed normal nerve fiber layer thickness, C/D was 0.33 in the right eye and 0.53 in the left eye. Ultrasonography showed that there were no special findings in vitreous cavity and posterior wall of the ball in both eyes, but the highly reflective band was separated from the wall in the periphery (Fig. 2). The axial length of the eye was 22.95 mm in the right eye and 23.09 mm in the left eye measured by Lenstar. Ultrasound biomicroscopy (UBM) (Suoer, CHN) showed bilateral ciliochoroidal effusions, disappearance of the ciliary sulcus, closure of the angle of the anterior chamber, and anterior displacement of the lens-iris diaphragm (Fig. 3). Treatment with timolol drops twice daily OU, atropine eye ointment once daily OU, oral methazolamide (50 mg) twice daily, and oral prednisone (30 mg) once daily. Syphilis, HIV and tuberculosis tests were all negative,

A day later, the patient’s uncorrected vision was 20/125 OU and was corrected to 20/50 OD and 20/40 OS with − 5.75D OU. IOP was 36 mmHg OD and 39 mmHg OS, the anterior chamber angles remained closed. On the third day, the patient’s IOP was 14 mmHg OD and 13 mmHg OS. On the fourth day, IOP was 8 mmHg OD and 9 mmHg OS. Treatment with oral and topical aqueous suppressants was terminated. On the fifth day, the uncorrected vision improved to 20/20 OU, and the myopia had disappeared. IOP was 11 mmHg OD and 12 mmHg OS. UBM revealed major resolution of the ciliochoroidal effusions in both eyes, deepening of the anterior chamber, return of the lens-iris diaphragm to a more posterior position. The anterior chamber depth of both eyes were 2.98 mm OD and 2.90 mm OS. The lens thickness was 4.12 mm OD and 4.06 mm OS. Half a year later, the follow-up UBM demonstrated complete resolution of the ciliochoroidal effusion. The anterior chamber depth was 2.79 mm OD and 2.91 mm OS.

## Discussion and conclusions

Although there has been a report of acute angle-closure glaucoma after taking cold medicines (the cold medicine component also had chlorphenamine), several important examinations such as UBM have not been performed and the refractive state of the eyes was not mentioned either in that report [5]. Therefore, it is not known whether there was a similar uveal effusion and a transient myopia. In another recent case report for bilateral simultaneous acute angle closure attack triggered by OTC flu medication, it showed symptoms and signs of typical angle-closure glaucoma, the patient’s axial length was shorter than normal and the refraction was hyperopia [6]. Our case is the first case to describe in detail the acute uveal effusion, angle-closure glaucoma, and transient myopia after oral taking OTC flu medication.

There have been several reports demonstrated that various types of drugs can induce acute uveal effusion with the performance of acute angle-closure glaucoma, such as diuretics, topiramate, sulphonamides, and acetazolamide et al. [1, 7, 8]. All reported cases have similar pathogenesis.

The OTC, oral cold and flu medication contained multiple ingredients that may cause the acute angle-closure glaucoma with uveal effusion. Paracetamol is one of the most popular and most commonly used analgesic and antipyretic drugs around the world [9]. It can affect the serotoninergic pathways which may cause the uveal effusion like many other drugs [1]. However, the pathophysiological mechanisms of drug-related transient myopia and acute angle-closure glaucoma are still unclear. How the implicated drugs produce choroidal swelling and bilateral angle closure glaucoma is currently unknown. In another review, the suggested etiologies postulated to be prostaglandin mediated and the most prevalent hypothesis is the hapten hypothesis that the reactive drug metabolites bind to the proteins in the the uveal tissue triggered an immune response [7, 10]. The effusion of the ciliary body and choroid leads to the anterior movement of the lens-iris diaphragm, forward rotation of ciliary body directly pushing the iris root forward, resulting in the closure of the angle of the anterior chamber, or even the closure of the Schlemm’s canal. In addition, the lenses become thicker caused by decreased zonular tension secondary to ciliary body edema and uveal effusion, which is also a factor for shallowing anterior chamber. The mechanism of transient myopia is not fully understood, but it may be that ciliary body swelling causes zonule relaxation, resulting in increased curvature of lens surface and spasm of accommodation, lens thickening and forward displacement.

If binocular acute angle closure glaucoma and myopia occur at the same time, it must be highly doubtful whether there is a history of relevant medication. Further evaluation of uveal effusion is needed by UBM. The primary treatment is to stop using related drugs. Followed by the reduction of intraocular pressure treatment, glucocorticoids may be helpful for recovery. Within a few days, symptoms and signs will be relieved and the prognosis is good.

We present a case of transient bilateral uveal effusions inducing acute angle-closure glaucoma and acute myopia after taking overdose non-prescription cold and flu medication. Although one case is not sufficient to prove the relationship between cold medicine and abnormal eye manifestations, it reminds us that the occurrence of such complications may be related to medicine. Due to the limitations of single case report, we still cannot rule out the coincidence between oral influenza drugs and ocular manifestations. However, due to the extensive use of over-the-counter flu drugs, ophthalmologists or emergency physicians and other primary physicians should be familiar with the situation discussed and aware of the diagnosis of acute angle-closure glaucoma associated with uveal effusion.

## Data Availability

All data are shown in the figures.

## Abbreviations

IOP: intraocular pressure
UBM: ultrasound biomicroscopy
